# Integration and relative value of biomarkers for prediction of MCI to AD progression: Spatial patterns of brain atrophy, cognitive scores, APOE genotype and CSF biomarkers^☆^^☆☆^

**DOI:** 10.1016/j.nicl.2013.11.010

**Published:** 2013-11-28

**Authors:** Xiao Da, Jon B. Toledo, Jarcy Zee, David A. Wolk, Sharon X. Xie, Yangming Ou, Amanda Shacklett, Paraskevi Parmpi, Leslie Shaw, John Q. Trojanowski, Christos Davatzikos, for the Alzheimer's Neuroimaging Initiative

**Affiliations:** a Section of Biomedical Image Analysis, Department of Radiology, and Center for Biomedical Image Computing and Analytics, University of Pennsylvania, Philadelphia, PA, USA; b Department of Pathology & Laboratory Medicine, Institute on Aging, Center for Neurodegenerative Disease Research, University of Pennsylvania School of Medicine, Philadelphia, PA, USA; c Memory Center, University of Pennsylvania, Philadelphia, PA, USA; d Department of Biostatistics and Epidemiology, University of Pennsylvania Perelman School of Medicine, Philadelphia, PA, USA

**Keywords:** Early Alzheimer's disease, Biomarkers of AD, Magnetic resonance imaging, Dementia, Mild cognitive impairment, Cerebrospinal fluid, Amyloid

## Abstract

This study evaluates the individual, as well as relative and joint value of indices obtained from magnetic resonance imaging (MRI) patterns of brain atrophy (quantified by the SPARE-AD index), cerebrospinal fluid (CSF) biomarkers, APOE genotype, and cognitive performance (ADAS-Cog) in progression from mild cognitive impairment (MCI) to Alzheimer's disease (AD) within a variable follow-up period up to 6 years, using data from the Alzheimer's Disease Neuroimaging Initiative-1 (ADNI-1). SPARE-AD was first established as a highly sensitive and specific MRI-marker of AD vs. cognitively normal (CN) subjects (AUC = 0.98). Baseline predictive values of all aforementioned indices were then compared using survival analysis on 381 MCI subjects. SPARE-AD and ADAS-Cog were found to have similar predictive value, and their combination was significantly better than their individual performance. APOE genotype did not significantly improve prediction, although the combination of SPARE-AD, ADAS-Cog and APOE ε4 provided the highest hazard ratio estimates of 17.8 (last vs. first quartile). In a subset of 192 MCI patients who also had CSF biomarkers, the addition of Aβ_1–42_, t-tau, and p-tau_181p_ to the previous model did not improve predictive value significantly over SPARE-AD and ADAS-Cog combined. Importantly, in amyloid-negative patients with MCI, SPARE-AD had high predictive power of clinical progression. Our findings suggest that SPARE-AD and ADAS-Cog in combination offer the highest predictive power of conversion from MCI to AD, which is improved, albeit not significantly, by APOE genotype. The finding that SPARE-AD in amyloid-negative MCI patients was predictive of clinical progression is not expected under the amyloid hypothesis and merits further investigation.

## Highlights

• 813 ADNI-1 subjects are analyzed using pattern recognition methods.
• Combination of SPARE-AD and ADAS-Cog offer high predictive index on MCI progression.
• Cox PH models showed predictors were highly associated with time to AD conversion.
• SPARE-AD in amyloid-negative MCI patients predicts clinical progression.

## 1 Introduction

Alzheimer's Disease (AD) is the most common form of dementia and a major health and socioeconomic concern (Hurd et al., 2013); therefore, early detection and disease modifying drug development are critically important. Mild cognitive impairment (MCI), in particular, has been an increasingly common target of potential therapeutic trials. However, the neuropathological substrates of MCI are heterogeneous (Schneider et al., 2009) and, despite the high rate of conversion to AD, a significant number of MCI patients remain stable (Petersen et al., 2009), or even revert to being cognitively normal (CN) (Manly et al., 2008). Developing predictors of an MCI individual's likelihood to progress clinically is therefore important. In addition to biomarkers of neurodegeneration (e.g. structural magnetic resonance imaging (sMRI)), the new research criteria for MCI incorporate the use of biomarkers of Aβ deposition to define MCI due to AD (Albert et al., 2011). Aβ deposition can be measured using PET tracers (Clark et al., 2012a; Ikonomovic et al., 2008) which correlate with decrease in Aβ_1–42_ in CSF (Fagan et al., 2009; Toledo et al., 2011). Both measures show a high accuracy for predicting AD neuropathology (Clark et al., 2012a; Shaw et al., 2009; Silverman et al., 2001; Toledo et al., 2012). CSF concentrations have shown promise in predicting conversion from MCI to AD (Hampel et al., 2010a, 2010b; Schuff et al., 2009; Shaw et al., 2009). However, when combined with other biomarkers, CSF has lower predictive power, especially compared to measures of brain atrophy (Davatzikos et al., 2011; Gomar et al., 2011; Vemuri et al., 2009a; Walhovd et al., 2010; Westman et al., 2012). It has been suggested that the presence of amyloid heightens the risk of conversion to AD, perhaps due to changes taking place in an early stage and followed by a ceiling effect (Jack et al., 2010a, 2013b). Alternatively, it is possible that there is another, non-causal, mechanism by which amyloid plaques and atrophy are related. These interpretations would be consistent with the relatively weak correlation between amyloid burden and cortical atrophy in regions typically associated with AD in cognitively normal individuals (Becker et al., 1996; Driscoll et al., 2009, 2011), and the similar amyloid levels between amnestic mild cognitive impairment (aMCI) and CN individuals, despite respective hippocampal volumes being different (Jack et al., 2008b), albeit some studies have shown stronger association between amyloid deposition and atrophy patterns (Tosun et al., 2011).

MRI-derived markers have been of central interest in characterizing brain structure in AD (Davatzikos et al., 2008a, 2008b, 2009; Fox and Schott, 2004; Jack et al., 2003; Kloppel et al., 2008; Schuff et al., 2009; Vemuri et al., 2009a; Wolz et al., 2011), and patterns of brain atrophy obtained from MRI have been shown to be relatively good predictors of conversion from CN to MCI (Davatzikos et al., 2008b, 2009; Driscoll et al., 2009; Vemuri et al., 2009b) and from MCI to AD (Adaszewski et al., 2013; Davatzikos et al., 2011; Eskildsen et al., 2012; Plant et al., 2010). The most commonly used sMRI biomarker is hippocampal volume, which is severely affected by AD (Fox et al., 1996; Jack et al., 1992, 2010b; Schuff et al., 2009). Hippocampal volumes alone, however, have limited accuracy for individualized diagnosis and prediction, as there is considerable overlap between hippocampal volumes of CN and AD individuals, and even more with MCI (Fan et al., 2008). As a result, hippocampal volumes do not capture the entire pattern of brain atrophy in AD or its prodromal stages (Dickerson and Wolk, 2012; Dickerson et al., 2009; Wolk et al., 2010).

Relatively recent literature has shown that pattern analysis methods are powerful diagnostic and predictive tools (Aksu et al., 2011; Costafreda et al., 2011; Davatzikos et al., 2009; Dickerson and Wolk, 2012; Duchesne et al., 2008; Hinrichs et al., 2009; Kloppel et al., 2008; Liu et al., 2004; McEvoy et al., 2009, 2011; Plant et al., 2010; Vemuri et al., 2009b; Wolz et al., 2011). One such index, the SPARE-AD score, calculated using a pattern classification method described in (Davatzikos et al., 2009; Fan et al., 2007), has been previously determined to be a good predictor of MCI to AD conversion (Misra et al., 2009) as well as of conversion from CN to MCI in healthy older adults (Davatzikos et al., 2008b, 2009).

Herein we present analysis of all ADNI-1 baseline images available by March 2013, and subsequently focus on a subset of MCI participants with at least 3-month, and up to 6-year clinical follow-up. We investigate the value of the SPARE-AD index in predicting 3-year stability from baseline scans, as well as its combination with APOE genotype, CSF biomarkers, and ADAS-Cog data. The main contributions of this work are 1) the analysis of 813 participants, providing a large number of subjects for the training and testing datasets and enabled us to establish the value of such pattern analysis methods as highly sensitive and specific imaging biomarkers of AD; 2) the combination of imaging, APOE genotype, CSF biomarkers, and ADAS-Cog allowed us to evaluate individual, as well as combined value of different types of AD biomarkers; 3) a longer follow-up using the larger cohort (mean follow-up time was 30 months),as opposed to most previous studies using ADNI. Our work largely builds upon the results of the study in Landau et al. (2010), where relative diagnostic and prognostic values of various AD biomarkers were examined on the same ADNI cohort. Our work is different in two respects: 1) we perform extensive survival analysis using data up to a 6-year follow-up period, instead of 1.9 years, thereby assessing the value of various biomarkers for predicting longer-term clinical stability; 2) we use the SPARE-AD score to capture spatial patterns of brain atrophy, which has been shown in several previous studies (and replicated herein) to offer high diagnostic and predictive value on an individual basis.

## 2 Material and methods

### 2.1 Subjects

Data from ADNI1 participants [www.adni-info.org] were used. All baseline images available for download on ADNI's website [adni.loni.ucla.edu] in pre-processed forms by March 2013 were included (232 CN individuals, 200 AD, and 381 MCI patients). Subject characteristics are summarized in Table 1.

### 2.2 MRI acquisition

Acquisition of 1.5-T MRI data at each performance site followed a previously described standardized protocol that included a sagittal volumetric 3D MPRAGE with variable resolution around the target of 1.2 mm isotropically. The scans had gone through certain correction methods such as gradwarp, B1 calibration, N3 correction, and (in-house) skull-stripping. See www.loni.ucla.edu/ADNI and Jack et al. (2008a) for details.

### 2.3 Collection and analysis of CSF biomarkers

CSF biomarker collection is described in detail under (www.adni-info.org/ADNIStudyProcedures.aspx). Briefly, lumbar puncture was performed with a 20-gauge or 24-gauge spinal needle as described in the ADNI procedures manual after written informed consent was obtained, as approved by the Institutional Review Board (IRB) at each participating center. Aβ_1–42_, total tau (t-tau) and tau phosphorylated at residue 181 (p-tau_181_) were measured in each of the 416 CSF ADNI baseline aliquots using the multiplex xMAP Luminex platform (Luminex Corp, Austin, TX) with Innogenetics (INNO-BIA AlzBio3, Ghent, Belgium; for research use only reagents) immunoassay kit-based reagents as described by (Shaw et al., 2009). Abnormal CSF levels were determined via a model combining t-tau, Aβ_1–42_ and p-tau_181p_ (Shaw et al., 2009) and pathological Aβ_1–42_ levels were considered to be levels below 192 pg/mL. AD-like CSF signature was described by (Shaw et al., 2009).

### 2.4 Image pre-processing

The images were processed with a freely-available pipeline (Davatzikos et al., 2001) (for software, see www.rad.upenn.edu/sbia). Briefly, images were segmented into 3 tissue types: gray matter (GM), white matter (WM), and cerebrospinal fluid (CSF). After a high-dimensional image warping to an atlas, regional volumetric maps for GM, WM and CSF were created, referred to herein as RAVENS maps. RAVENS maps are used for voxel-based analysis and group comparisons of regional tissue atrophy, as well as for constructing an index of AD brain morphology.

### 2.5 The SPARE-AD index as morphologic phenotype of AD

SPARE-AD has been extensively described elsewhere (Davatzikos et al., 2009; Fan et al., 2007). For SPARE-AD computation, the method looks for the combination of brain regions, which can form a unique pattern that maximally differentiates between AD and CN and then trains a nonlinear support vector machine (SVM) model that measures this pattern. This model is then evaluated on a new scan: positive values indicating presence of AD-like characteristics and negative values conversely. After determining the classifier that separates AD/CN, this classifier was applied to baseline MCI patients' scans, thereby providing SPARE-AD scores. Although our previous analyses have reported the SPARE-AD score using smaller samples, which had been trained on data from 66 CN individuals and 56 AD patients, all ADNI participants (Fan et al., 2008), we retrained the same algorithm on this significantly larger set of data from 232 CN subjects and 200 AD patients, in order to obtain the best possible stability and generalization potential. SPARE-AD scores were also derived for the CN and AD individuals. However, since these individuals were part of the model's building, their scores were derived using 10-fold cross-validation (10% of the data was left out for the outer loop/test set for testing and assessing the area under the curve (AUC) of the receiver operating characteristic (ROC) curve, the rest was treated as the training set; parameters were optimized in this 90% of the sample by splitting it into training and validation datasets, using leave-one-out and a parameter grid-search; optimized SVM parameters included kernel size and slackness parameter (C); optimized models were applied exactly as determined from the training set to the remaining 10%, and classifications were recorded. This procedure was repeated 10 times, so that each sample gets a classification score).

### 2.6 Statistical methods

In our survival analysis, we included 381 MCI subjects (mean follow-up time = 30 months, SD = 18.6, 25th percentile 12 months, median 24 months, 75th percentile 48 months). To perform the survival analysis of various combinations of markers, we utilized a separate linear support vector machine (SVM) (Vapnik, 1998) trained (implemented in weka public domain software (Hall et al., 2009)) using a combination of SPARE-AD scores and other relevant markers such as ADAS-Cog, APOE ε4 and CSF biomarkers. This is independent of the SVM trained in the algorithm used for generating SPARE-AD scores. We chose the SVM's slackness parameter (C) using cross-validation while training the classifier on AD and CN; the optimized classifier was then applied to the (separate) MCI set, providing a continuous index between 0 and 1 which was used as a predictor in the survival analysis. Using this continuous index as a predictor, we compared the magnitudes of the association between predictors and time to conversion from MCI to AD using Cox proportional hazards models. Cox models were used: 1) treating the predictor as a continuous measure, and 2) splitting the predictor into quartiles. To compare across models, each of the predictors was standardized by subtracting its mean and dividing by its standard deviation. In a subset of subjects (192 MCI patients, 100 converted to AD) who also had CSF biomarkers, the aforementioned survival analysis was repeated, albeit now considering combinations of markers including CSF biomarkers. For each pair-wise comparison, we tested for differences in the effects of two predictors using the cross-model testing method described by Weesie (Weesie, 1999) with Cox proportional hazards models on time to conversion from MCI to AD. Besides the two predictor values for each subject, the cross-model testing procedure requires us to include the observed survival time twice for a given subject in the Cox model. Since each pair-wise comparison model included two correlated outcomes per subject from each of the two predictors, robust sandwich-type estimators to account for clustering (correlation) within subject were used to estimate variances. Wald tests were used to test for significant differences, which would indicate that the two predictors had significantly different hazard ratios (HR) of time to conversion. Finally, Kaplan–Meier survival function estimates were plotted using quartiles of each predictor. All Cox models were adjusted for age, gender, and education covariates. All statistical tests were two-sided. Statistical significance was set at < 0.05 level. Statistical analyses were conducted using STATA version 12.0 (StatCorp; College Station, TX) software.

## 3 Results

### 3.1 SPARE-AD as an MRI marker of AD

**Fig. 1:**
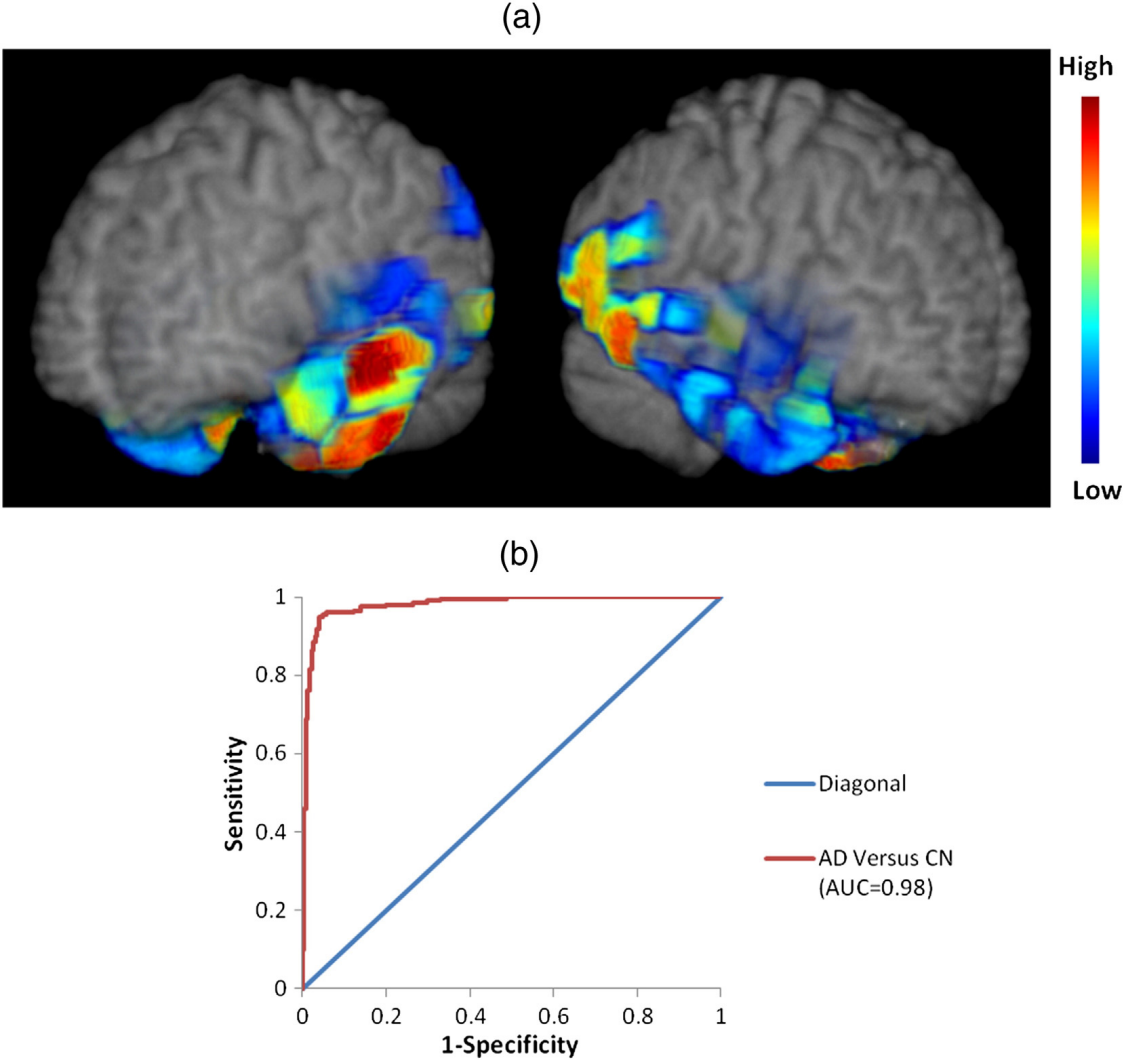
(a) Visualization of the regions used to build the SPARE-AD index, when all 3 (GM, WM and brain CSF) RAVENS maps were used jointly. (Left) Temporal lobe and hippocampus of the left hemisphere; (right) temporal lobe and hippocampus of the right hemisphere. Images are in radiology convention. The color scale is graded (low to high) based on relevance of different brain regions for classification into AD/CN, herein measured by the frequency by which a region was selected by the 10 models produced by the 10-fold cross-validation. (b) ROC curve and performance graph of AD and CN classification results using GM, WM and brain CSF tissue density maps, obtained via fully cross-validated procedures. (For interpretation of the references to color in this figure legend, the reader is referred to the web version of this article.)

The best MRI-based diagnostic accuracy was achieved by jointly considering the RAVENS maps of GM, WM and CSF, thereby forming a SPARE-AD score by evaluating regional patterns of atrophy and ventricular enlargement. 3D visualizations (Fig. 1) help appreciate brain regions participating in the diagnostic model (temporal horn and hippocampal regions are not directly visible). Many temporal lobe brain regions, as well as CSF regions largely being part of the temporal horn of the ventricles, were used for evaluating the spatial pattern of brain atrophy and ventricular expansion that was most distinctive of AD patients. The 10-fold cross-validated ROC curve obtained using the SPARE-AD score, is also shown in Fig. 1.

### 3.2 MCI survival analysis

**Fig. 2:**
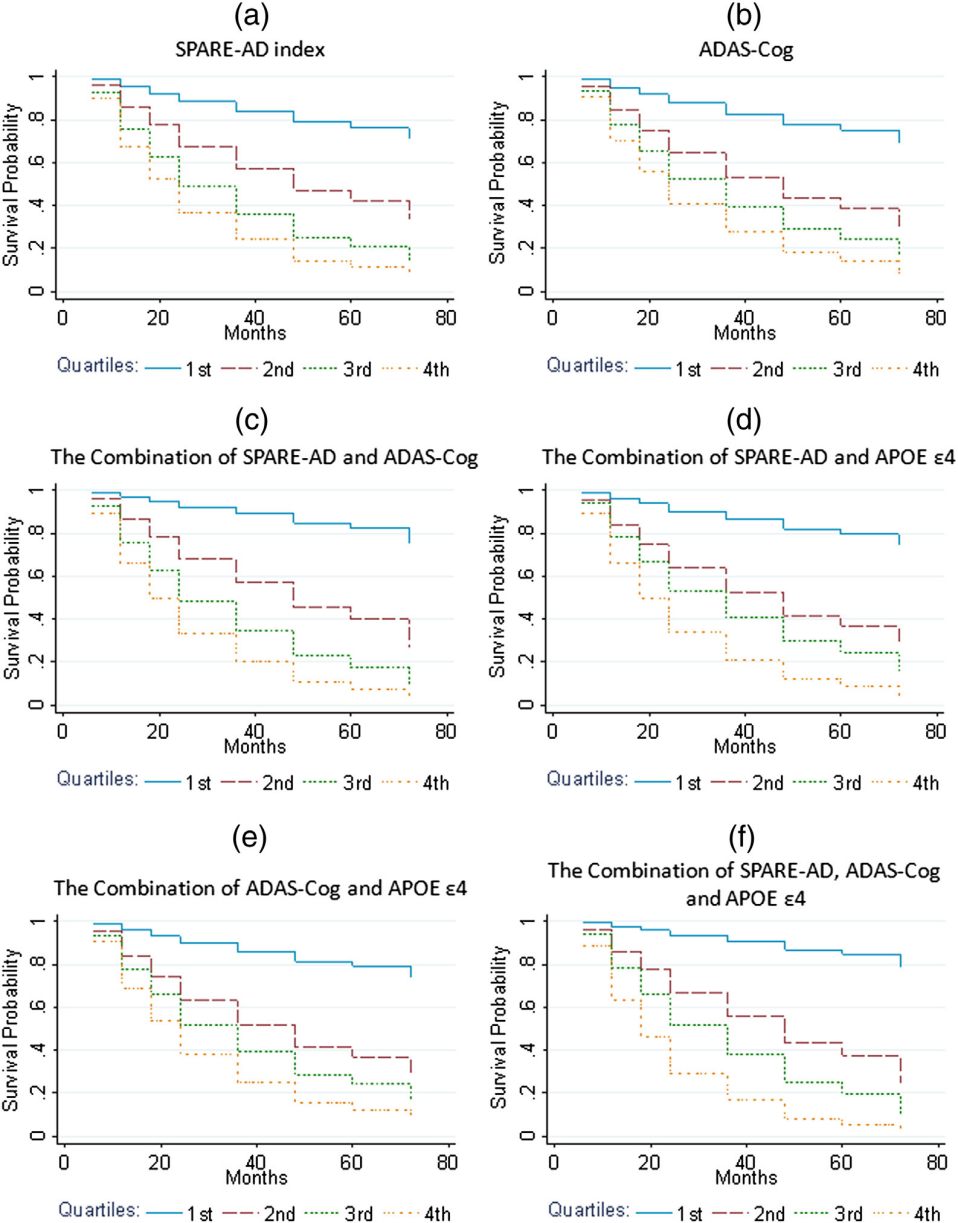
Survival curves for (a) SPARE-AD index alone; (b) ADAS-Cog alone; (c) the combination of SPARE-AD and ADAS-Cog; (d) the combination of SPARE-AD and APOE ε4; (e) the combination of ADAS-Cog and APOE ε4, and (f) the combination of SPARE-AD, ADAS-Cog and APOE ε4.

Survival curves for the SPARE-AD index alone, ADAS-Cog alone, the combination of SPARE-AD and ADAS-Cog, the combination of SPARE-AD and APOE ε4, the combination of ADAS-Cog and APOE ε4, and the combination of SPARE-AD, ADAS-Cog and APOE ε4, all in quartiles, are shown in Fig. 2. The plots show that those in the 1st (lowest) quartile of predictor values have the lowest risk of conversion from MCI to AD at any given time, and for higher quartiles, the risk of conversion at any given time increases. Furthermore, compared to predictors based on individual markers, predictors based on a combination of markers show greater separation of survival curves, particularly of the 1st quartile from other quartiles. MCI subjects had variable follow-up length (mean = 30 months, SD = 18.6, median = 24 months): out of 381 MCI subjects, 188 progressed to AD (mean = 23 months, SD = 14.5, median = 18 months). All of the 193 subjects who did not develop AD were considered right-censored at last follow-up and included in the analysis. Adjusted associations between different combinations of markers and time from MCI to AD conversion are shown in Table 2. For each predictor, adjusted hazard ratios (HR) from two Cox models are shown: 1) treating predictor as continuous, and 2) splitting predictor into quartiles. The HR for continuous measures represent the risk of converting to AD from MCI at any given time point for a one unit increase in the predictor value, given that age, gender, and education are held constant. For models using quartiles, the reference group is the 1st quartile. All predictors have a significant (p < 0.001) association with time to conversion from MCI to AD. As the value of each predictor increases, the hazard of conversion increases, keeping age, gender, and education constant. The p-values from the tests comparing the different predictors across models are shown in Table 3. There was no significant difference (p = 0.865) between the adjusted HR of time to conversion from SPARE-AD (HR = 2.2) and the adjusted HR from ADAS-Cog (HR = 2.0). The combination of SPARE-AD and ADAS-Cog was better than either of the individual models in predicting time to conversion (each p < 0.001). The inclusion of APOE ε4 to SPARE-AD significantly improved prediction of time to conversion (p < 0.001), whereas the inclusion of APOE ε4 to ADAS-Cog did not yield significant improvement (p = 0.491). Compared to the prediction of time to conversion based on the combination of SPARE-AD and ADAS-Cog, the inclusion of APOE ε4 presence did not significantly improve prediction (p = 0.638). The analogous survival analysis in the smaller sample also having CSF biomarkers is presented in Tables 4 and 5. Based on the comparison between the models (Table 5), adding APOE ε4, CSF, or the combination of both markers did not significantly improve any predictions of time to conversion.

### 3.3 SPARE-scores in MCI stratified by CSF Aβ_1–42_

**Fig. 3:**
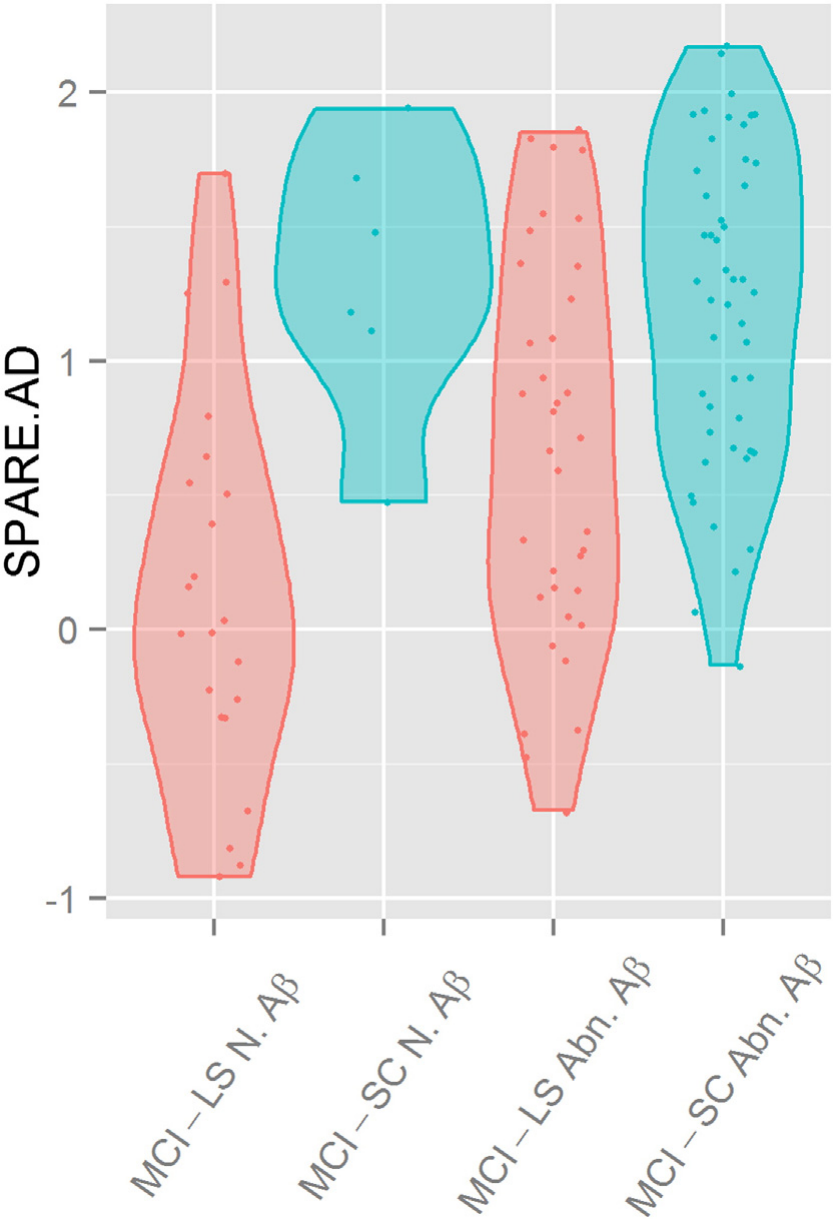
Violin plot depicting baseline SPARE-AD scores stratified by clinical diagnosis, MCI-SC (blue) and MCI-LS (red), and presence or absence of AD-like CSF Aβ_1–42_ values. (For interpretation of the references to color in this figure legend, the reader is referred to the web version of this article.)

**Fig. 4:**
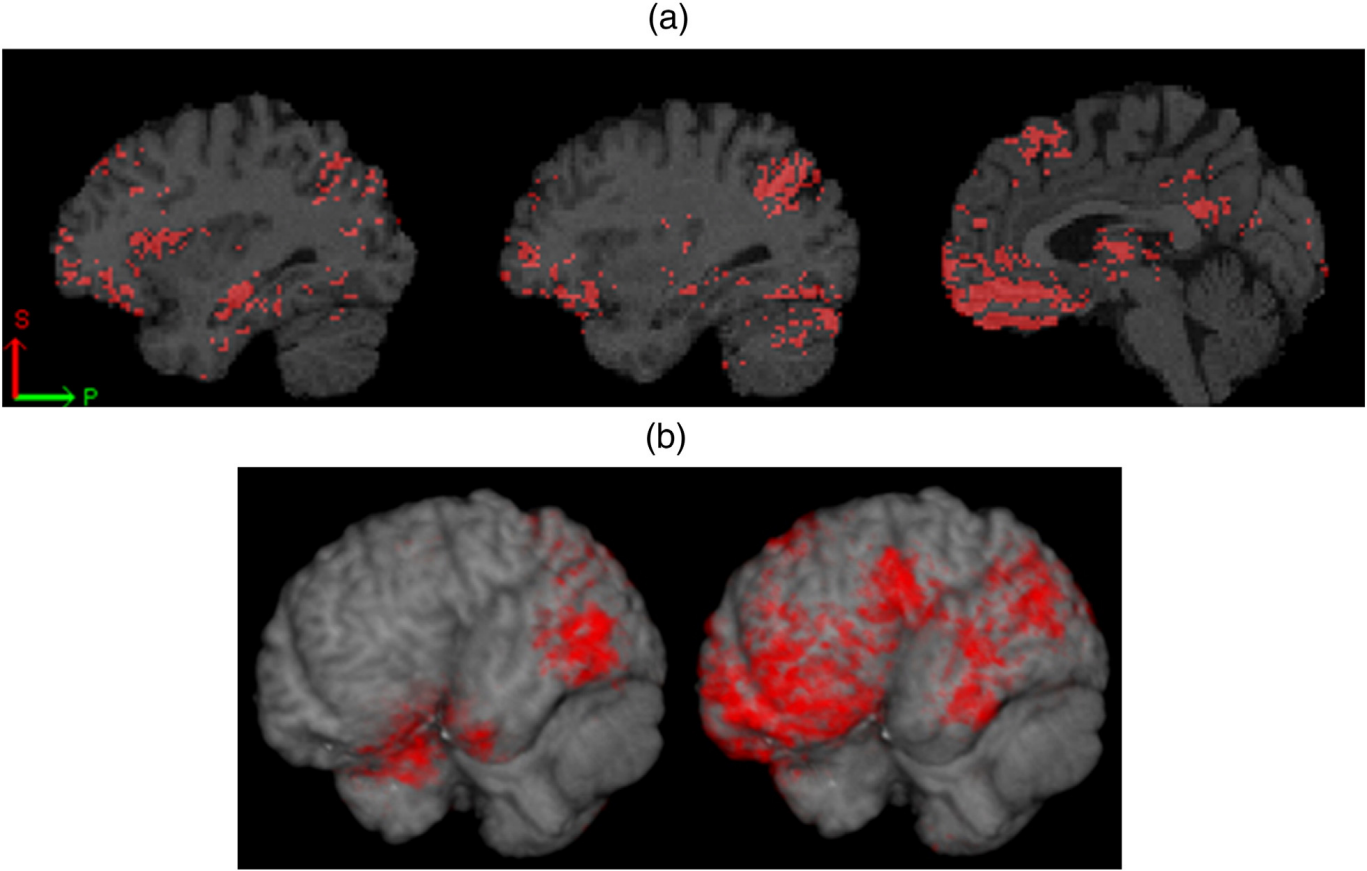
(a) Maps of the p value produced by optimally-discriminative voxel-based analysis (ODVBA) (Zhang and Davatzikos, 2011) showing differences between MCI-LS and MCI-SC based on the normal Aβ_1–42_ subsample. Significantly more GM atrophy for hippocampus, prefrontal lobe and precuneus in MCI-SC relative to MCI-LS. The maps were thresholded at the p = 0.01 level. (b) 3D renderings of statistically significant differences between MCI-LS and MCI-SC. normal Aβ_1–42_ subsample (right); pathological Aβ_1–42_ subsample (left). The maps were thresholded at the p = 0.01 level.

Finally, we studied the relationship between AD-like CSF signature (Shaw et al., 2009) and longitudinal clinical diagnosis with SPARE-AD. In order to evaluate the relationship between brain atrophy and amyloid burden, the values of SPARE-AD were examined in a subset of MCI individuals who either converted to AD within at most 18 months (short converters, MCI-SC) or remained stable for at least 36 months (long term stable, MCI-LS). In particular, out of this subset of MCI patients (MCI-LS plus MCI-SC), 28 (6 MCI-SC and 22 MCI-LS) had normal Aβ_1–42_ levels (> 192 pg/mL) and 84 (48 MCI-SC and 36 MCI-LS) had pathological Aβ_1-42_ levels (≤ 192 pg/mL). We tested if the SPARE-AD score was associated with the presence of pathological CSF values or the longitudinal clinical diagnosis using a linear regression analysis. Both MCI-SC clinical diagnosis (t = 4.96, p < 0.0001) and AD-like CSF Aβ_1–42_ levels (t = 2.34, p = 0.02) were associated with higher SPARE-AD scores. Having a MCI-SC diagnosis (Beta = 0.65) was associated with a larger effect size than the presence of low Aβ_1–42_ levels (Beta = 0.36) (Fig. 3). There was no interaction between clinical and CSF group (t = − 1.92, p = 0.058). Mean group values are presented in Table 6; SPARE-AD values were significantly different between MCI-LS and MCI-SC which had normal Aβ_1–42_ levels, underlying the high predictive value of SPARE-AD in this amyloid-negative group. Nevertheless, subjects with normal Aβ_1–42_ levels showed distinct changes compared to those with pathological levels (Fig. 4(a)). 3D renderings of group differences between MCI-LS and MCI-SC are shown in Fig. 4(b) for both the positive and the negative amyloid groups.

## 4 Discussion

The present study evaluated the integration and relative value of spatial patterns of brain atrophy (SPARE-AD index), CSF biomarkers, measures of cognitive performance (ADAS-Cog), along with APOE genotype, in predicting the individual risk of converting from MCI to AD. Moreover, the value of SPARE-AD as an MRI-derived marker of AD-like atrophy was further investigated in a cohort of CN individuals and AD patients, and was found to display excellent sensitivity and specificity in classifying AD patients, with a cross-validated AUC of 0.98 in the hold-out test set. As baseline predictors of conversion to AD, SPARE-AD and ADAS-Cog were of similar predictive value, and their combination significantly improved the ability to predict risk of conversion to AD (Hazard ratio of 13.6 between top and bottom quartiles) compared with either of the predictors alone. Adding APOE genotype to the combination of SPARE-AD and ADAS-Cog further improved the predictive ability (Hazard ratio of 17.8 between top and bottom quartiles), albeit the improvement was not statistically significant. This is consistent with APOE ε4 being a risk factor for AD, however its value for individual patient predictions is limited (Aguilar et al., 2013; Foster et al., 2013). CSF offered marginal improvement to predictive power, which was not statistically significant (Vemuri et al., 2009a; Walhovd et al., 2010; Westman et al., 2012).

Our survival analysis complements similar analyses (McEvoy et al., 2011; Vemuri et al., 2009b), yet obtains better baseline-based prediction using the combination of SPARE-AD and ADAS-Cog. Our results also complement several studies that used a specific follow-up time as cut-off for dichotomous conversion/stability outcome (Aksu et al., 2011; Fan et al., 2008; Kloppel et al., 2008; Plant et al., 2010; Vemuri et al., 2008, 2009b), albeit those results are not directly comparable to ours as we do not have such dichotomous classification depending on some pre-defined and somewhat arbitrary length of conversion time (Hinrichs et al., 2011; Plant et al., 2010).

The relatively limited value of CSF biomarkers alone, especially of Aβ_1–42_, in predicting clinical progression could be argued to reflect a potential ceiling effect in amyloid deposition in the brain in early disease stages (Fleisher et al., 2012; Jack et al., 2010a, 2013a, 2013b; Toledo et al., 2013c), beyond which actual amyloid levels do not have predictive value, whereas subsequent atrophy is a better predictor. Alternatively, other neurodegenerative and vascular conditions in addition to amyloid plaque deposition can potentially account for the cognitive symptoms in MCI patients with normal Aβ_1–42_ and p-tau_181_ values (Schneider et al., 2009). Importantly, the predictive value of amyloid might be higher during early disease stages, which underlines the need for building dynamic imaging markers in AD, since predictive value of various markers is likely to depend on disease stage. The lack of additive value for the tau markers over SPARE-AD is somewhat expected, as tau levels and brain atrophy tend to correlate well (Toledo et al., 2013b), and potentially MRI-derived SPARE-AD index more directly captures neurodegeneration. However, one might have expected higher predictive value of tau markers alone, relatively to what we found.

An intriguing finding of our study was that in amyloid-negative MCI patients, positive SPARE-AD values were predictive of conversion indicating that SPARE-AD captures a pattern of atrophy that characterizes clinical AD cases and is able to predict clinical changes in amnestic MCI subjects with a non AD-like CSF signature. In particular, AD-like patterns of brain atrophy were more pronounced in MCI-SC relative to MCI-LS (p = 0.0008), and included regions such as the precuneus, which show early changes in AD. This finding adds to a number of recent findings that indicate that considerable percentage of both cognitively normal older adults (Driscoll et al., 2011; Wirth et al., 2013) and preclinical AD (Jack et al., 2012) have atrophy in regions affected by AD without the presence of amyloid. An extensive review of the literature on the relationship between amyloid burden, AD-like brain atrophy and cognitive function can be found in (Fjell et al., 2010), where considerable concerns about the widely accepted amyloid hypothesis, and therefore about the utility of amyloid markers in predicting clinical progression, are discussed based on a number of findings from the literature. However, this finding can potentially be due to false negatives in the Luminex platform, i.e., assumed amyloid-negative individuals might actually have amyloid, or due to the presence of a different neurodegenerative mechanism with similar pattern of atrophy and clinical manifestation as AD. Moreover, the number of amyloid-negative MCI individuals was small, hence these findings should be replicated in a larger sample. Longitudinal studies in cognitively normal older adults are necessary to elucidate potential dynamic interplays and causal relationships between amyloid deposition, neuronal death, and cognitive decline, or perhaps to discover other mechanisms that lead independently to both amyloid deposition and neuronal death.

The spatial pattern of brain atrophy that differed between MCI-SC and MCI-LS (Fig. 4) was in agreement with other literature in the field using analogous methods (Whitwell et al., 2008). However, in addition to temporal and posterior parietal regions, our study identified significant prefrontal and orbitofrontal atrophy, especially in amyloid-negative subsample. 10% or more of the cases with a clinical diagnosis of AD do not have an underlying AD when assessed in neuropathological studies (Nelson et al., 2012; Toledo et al., 2012) and this percentage increases in the MCI stage. Because CSF biomarkers show a good correlation with AD pathology in the brain (Tapiola et al., 2009), it is possible that some amyloid-negative MCI individuals have a frontotemporal lobar degeneration and therefore these patients can show a different pattern of atrophy. This would be in agreement with independent studies comparing AD and frontotemporal dementia patients (Davatzikos et al., 2008c; McMillan et al., 2013). In addition, several different pathologies can be present in a single subject as we recently described in a small subset of ADNI subjects that came to autopsy that coincident pathologies are a common finding (Toledo et al., 2013a).

The predictive value of SPARE-AD in MCI individuals complements earlier studies that found similar AD-like patterns of brain atrophy being predictive of cognitive decline in cognitively normal older adults (Clark et al., 2012b; Dickerson and Wolk, 2012). Particularly relevant is our previous study (Clark et al., 2012b), since it used the exact same image analysis and SPARE-AD index. In that prospective longitudinal study of aging over an 8-year period, the rate of change of SPARE-AD was highly predictive of conversion from cognitively normal to MCI, with a cross-validated AUC of 0.89. These patterns of brain atrophy are therefore likely to progress slowly, yet steadily, many years before they eventually lead to MCI and then to dementia. Methods for capturing such relatively complex atrophy patterns, and combining them with measures of cognitive decline, are therefore important biomarkers of very early AD, potentially at stages in which interventions might be more effective.

## 5 Conclusion

We found that SPARE-AD, which quantifies spatial patterns of brain atrophy using pattern classification, was a highly sensitive and specific imaging marker of AD (cross-validated AUC = 0.98 in a cohort of 432 AD/CN individuals). Moreover, combination of SPARE-AD, ADAS-Cog and APOE genotype provided excellent predictive value in a cohort of 381 MCI individuals followed for a variable period of up to 6 years (HR = 17.8 between top and bottom quartiles), albeit the additive value of APOE ε4 presence was not statistically significant over the combination of SPARE-AD and ADAS-Cog. In addition to having implications for enrollment in clinical trials, these findings are becoming increasingly important in clinical settings where a variety of biomarkers are available. Thus, being able to provide prognostic information, including the timeframe of potential change, is of obvious importance in discussions with patients. Finally, the present findings related to CSF Aβ_1–42_ negative MCI patients also speak to questions concerning the proposed cascade of biomarker change and the pathophysiologic process of AD. Longitudinal studies starting relatively earlier in life would be necessary for deeper understanding of the dynamics of AD progression.

## Data and software availability

All SPARE-AD scores used herein have been uploaded to http://adni.loni.ucla.edu/. All image processing software used to derive SPARE-AD, importantly the DRAMMS deformable registration and COMPARE classification pipelines, are freely available for download under http://www.rad.upenn.edu/sbia, and involve fully automated procedures.

## Disclosure statement

The authors disclose no actual or potential conflicts of interest. Written informed consent was obtained for participation in these studies, as approved by the Institutional Review Board (IRB) at each participating center.

## Figures and Tables

**Table 1:** Characteristics of ADNI1 subjects included in the study.

	AD	CN	MCI
Subjects, n	200	232	381
Average age	75.6 ± 7.72	76.0 ± 5.01	74.8 ± 7.32
Gender (male/female)	103M, 97F	120M, 112F	244M, 137F
Average MMSE	23.3 ± 2.05	29.1 ± 1.00	27.0 ± 1.78
Average modified ADAS-Cog (85 point)	28.0 ± 9.51 (188)	9.5 ± 4.19 (229)	18.5 ± 6.64
Percentage having APOE ε4 alleles	66.0% (188)	26.6% (229)	54.1%

Parentheses show the subjects for which both ADAS and APOE ε4 alleles were available. AD = Alzheimer's disease dementia; APOE = apolipoprotein E; CN = cognitively normal; MCI = mild cognitive impairment; MMSE = Mini mental state examination; modified ADAS-Cog = the modified Alzheimer's Disease Assessment Scale, cognitive subscale.

**Table 2:** Hazard ratios of MCI to AD progression by standardized predictors in 381 MCI individuals.

	SPARE-AD			ADAS			SPARE-AD + ADAS			SPARE-AD + APOE ε4			ADAS + APOE ε4			SPARE-AD + ADAS + APOE ε4		
	HR	95% CI	p	HR	95% CI	p	HR	95% CI	p	HR	95% CI	p	HR	95% CI	p	HR	95% CI	p
Continuous	2.2	(1.8,2.6)	< 0.001	2.0	(1.7,2.4)	< 0.001	2.8	(2.2,3.6)	< 0.001	2.6	(2.0,3.2)	< 0.001	2.1	(1.7,2.4)	< 0.001	2.9	(2.2,3.6)	< 0.001
Quartiles			< 0.001			< 0.001			< 0.001			< 0.001			< 0.001			< 0.001
2nd quartile	3.2	(1.8,5.5)		3.3	(1.9,5.8)		4.7	(2.5,8.9)		4.4	(2.5,7.8)		4.3	(2.4,7.7)		5.8	(3.0,11.3)	
3rd quartile	5.8	(3.4,9.8)		4.9	(2.9,8.4)		9.0	(4.8,16.6)		6.2	(3.5,10.9)		6.1	(3.4,10.8)		9.7	(5.0,18.7)	
4th quartile	8.1	(4.7,14.0)		6.7	(4.0,11.5)		13.6	(7.3,25.2)		10.6	(5.9,18.9)		9.0	(5.1,15.8)		17.8	(9.2,34.4)	

**Table 3:** p-Values comparing magnitudes of association between (continuous) predictor and outcome using 381 MCI individuals.

	SPARE-AD	ADAS	SPARE-AD + ADAS	SPARE-AD + APOE ε4	ADAS + APOE ε4	SPARE-AD + ADAS + APOE ε4
SPARE-AD		0.865	< 0.001	< 0.001	0.873	< 0.001
ADAS	0.865		< 0.001	0.052	0.491	< 0.001
SPARE-AD + ADAS	< 0.001	< 0.001		0.209	0.002	0.638
SPARE-AD + APOE ε4	< 0.001	0.052	0.209		0.078	0.128
ADAS + APOE ε4	0.873	0.491	0.002	0.078		< 0.001
SPARE-AD + ADAS + APOE ε4	< 0.001	< 0.001	0.638	0.128	< 0.001	

**Table 4:** Hazard ratios of MCI to AD progression by standardized predictors using subset of 192 with CSF.

	SPARE-AD + ADAS			SPARE-AD + ADAS + APOE ε4			SPARE-AD + ADAS + CSF			SPARE-AD + ADAS + APOE ε4 + CSF		
	HR	95% CI	p	HR	95% CI	p	HR	95% CI	p	HR	95% CI	p
Continuous	2.5	(1.7,3.4)	< 0.001	2.5	(1.8,3.5)	< 0.001	2.7	(1.9,3.8)	< 0.001	2.6	(1.8,3.7)	< 0.001
Quartiles			< 0.001			< 0.001			< 0.001			< 0.001
2nd quartile	2.9	(1.3,6.4)		3.2	(1.4,7.1)		3.5	(1.6,7.7)		4.6	(2.1,10.2)	
3rd quartile	5.2	(2.4,11.4)		5.2	(2.4,11.6)		5.6	(2.6,11.9)		5.8	(2.6,12.8)	
4th quartile	8.7	(4.0,18.8)		10.8	(4.9,23.8)		9.3	(4.4,19.9)		11.5	(5.2,25.4)	

**Table 5:** p-Values comparing magnitudes of association between (continuous) predictor and outcome, using subsample with CSF available.

	SPARE-AD + ADAS	SPARE-AD + ADAS + APOE ε4	SPARE-AD + ADAS + CSF	SPARE-AD + ADAS + APOE ε4 + CSF
SPARE-AD + ADAS		0.533	0.205	0.271
SPARE-AD + ADAS + APOE ε4	0.533		0.229	0.271
SPARE-AD + ADAS + CSF	0.205	0.229		0.400
SPARE-AD + ADAS + APOE ε4 + CSF	0.271	0.271	0.400	

**Table 6:** SPARE-AD values were significantly different between MCI-SC and MCI-LS both for the Aβ_1–42_-normal MCI patients (top; p = 0.0008) and for Aβ_1–42_-pathological MCI patients (bottom; p = 0.0005).

	SPARE-AD mean (St dev)	
	MCI-SC	MCI-LS
Aβ_1–42_ > 192 pg/mL (normal)	1.31 (0.51)	0.13 (0.71)
Aβ_1–42_ < 192 pg/mL (pathological)	1.20 (0.59)	0.67 (0.72)
